# Automated medium–pressure modular liquid–gas reactor

**DOI:** 10.1155/S1463924689000362

**Published:** 1989

**Authors:** Catherine Porte, Fadl Hamdan, Alain Delacroix

**Affiliations:** Conservatoire National des Arts et Métiers 292 rue Saint Martin Paris 75141 France


Journal of Automatic Chemistry Vol. 11, No. 4 (July–August 1989), pp. 168–173

### Automated medium-pressure modular liquid–gas reactor

**Catherine Porte, Fadl Hamdan and Alain Delacroix**
*Conservatoire National des Arts et Métiers, 292 rue Saint Martin, 75141 Paris, France*

In the development laboratory, most liquid–gas reactions are carried out at low pressure because of the equipment customarily used. High-pressure reactions involve special equipment such as a steel reactor and a complex stirring system.

This paper describes an automated reactor in which reactions can be carried out using at least one gaseous reagent and one liquid reagent at a pressure of up to 5 bar. The reactor was used to produce a hydrogenation reaction, in which system all operations, i.e. introduction of liquids and gases, control and regulation of pressure, temperature and volume of gases introduced and finally draining of the reactor, were to be automated. The system is conceived in modular form, each module being responsible for one specific task [1,2].

First, we shall describe the modules which form the operative form.

### The Operative Part

The reactor, with a capacity of 1000 ml, made of Pyrex glass, is double jacketed and has a valve at its base. Three baffles are placed symetrically to eliminate vortex effects. The seal between the reactor and the lid is assured by a Viton diaphragm. Introduction of the liquid and gaseous reagents is carried out through tubes attached to the lid. All components are made of stainless steel. The different connecting pieces to the reactor make the apparatus adaptable. A spring-loaded safety valve is adjustable from the exterior. The opening pressure of the valve is only sensitive to pressure at the inlet (and is not affected by back-pressure). In order to adapt the opening pressure of the valve to the reaction being carried out, it is necessary only to adjust the compression of the spring valve.

Draining of the reactor is effected by gravitation in the case of non-loaded solutions; in the presence of solid particles (catalyst), discharge by suction through a stainless-steel tube immersed in the reactor is preferable. To avoid leaks, the drive of the stirring rod is magnetic. The shaft is fixed to an interior magnet and driven on all bearings. These two elements are situated in a pressure-tight chamber. The exterior magnet, driven by an electric motor and flexible drive shaft, operates outside the pressure chamber. The rotation of the exterior magnet drives the interior magnet by magnetic coupling.

The liquid reagents are introduced into the reactor by weight. This requires a balance as a sensor and a system for comparison. The introduction system consists of an electromagnet metering pump with a counter pressure valve and a bistable pneumatic valve. This also makes it possible to introduce reagents into the pressurized reactor during the course of a reaction. A digital comparator, connected in parallel to the analogue output of the balance, allows control of the amounts of any reagents to be introduced. The comparator establishes a connection between the analogue sizes measured and the pre-established thresholds, and provides the programmable controller with the corresponding logical signals.

The introduction of gases and the purge are carried out using a set of four two-way electrovalves: one normally closed for the introduction of the reactive gas (hydrogen, oxygen, etc.); one normally closed for the introduction of the inert gas (nitrogen); one normally open to isolate the reactor from the remainder of the installation if necessary; and one normally closed, linked to an aspirator, to create a vacuum in the reactor.

The measure and pressure regulation module consists of an analogue pressure sensor and a digital conditioner. This apparatus makes it possible to process the signals obtained by the pressure sensor. After reading and conditioning the analogue signals, it displays the value of the absolute pressure and provides, at its output, an analogue voltage of 0–10 V. The conditioner also provides the power supply for the sensor at 10 V a.c. or d.c.

The temperature inside the reactor is regulated by circulation of a heat-exchange fluid in a loop which includes the double jacket and an exterior bath in which heating is carried out by a circulation thermostat. With a cryoplunger for cooling, this thermostatic bath can operate at temperatures ranging between −20 and +120 °C, taking into account that of the reactive medium. Regulation of the bath temperature should take into account that of the reactive medium. Therefore, we used the setup shown in figure 1.

The circulation thermostat receives an external threshold, which varies with time, from a program transmitter. The evolution of this threshold takes place through several stages. The analogue input signal is produced by the programme transmitter. This apparatus, based on a microprocessor, is mainly intended to generate a signal, analogue or digital, variable with time, to command the threshold of the electronic regulator. It can also generate logical states in terms of time.

The control module for the measurement of flow and volume of gas introduced consists of a mass flow meter, a three-way electrovalve and a digital integrator. The mass flow meter consists of two elements: a flow sensor designed to measure an instantaneous gas flow and an

0142–0453/89 $3.00 © 1989 Taylor & Francis Ltd.

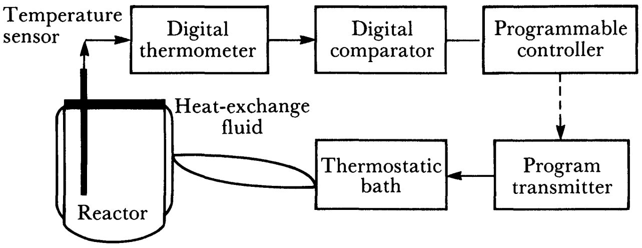

*Figure 1. Setup of the reactor system.*

electronic integrated circuit, transmitting a signal corresponding to a measured flow and permitting remote reading.

The digital integrator is designed to be included in a reactive process using a gaseous reagent. Coupled with the mass flow meter, it assures flow measurement and calculation of the volume of gas used, at normal temperature and pressure, during the reaction by integration. It can be programmed and used either locally or remotely by a programmable controller. The digital integrator is controlled by a ROM program. The parameters imposed by the user are retained in the RAM, safeguarded by a battery and are thus preserved in the event of a mains power failure. It also supplies the flow sensor in series +15 V.C.C./−15 V.C.C. from the mains voltage and arranges the analogue signal (voltage from 0 to 5 V) supplied by the mass flow meter. The signal is digitalised with the help of a voltage–frequency convertor associated with the counters.

The integrator also possesses the following components: a logical input triggering the start of integration by an external apparatus, e.g. a controller; a logical input 'Security', informing the apparatus of an outside event (e.g. closure order of the electrovalve for gas admission), and this signal is used to halt integration, after a period of 30 s, so as to free itself from the possible displacement of the zero on the mass flow meter for a zero flow: an analogue output 'Flow' allowing the signal supplied by the mass flow meter to be recorded; an analogue output 'volume' to record the evolution of this parameter; a logical output 'Security' activated when a threshold is reached (threshold of flow, volume or time of reaction); a logical output 'End of reaction' activated when the threshold 'time or volume' is reached; and an input/output RS 232 C allowing programming and editing on another computer facility.

The principle of integration is as follows. At time $t = 0$, the counters are set to zero and counting is started. There is then an accumulation of impulses delivered by the voltage–frequency convertor. After 1 s, the value shown in the counters is representative of the volume introduced, and therefore of the average flow during the same time. The order to start integration is supplied by the controller. The integrator takes into account the signals delivered by the mass flow meter when the electrovalve for gas admission is open, and for 30 s after its closure.

When overstepping at temporary thresholds occurs (flow, volume or reaction time), the integrator sends a logical signal to the controller, which then orders the closure of the electrovalve. Only one threshold 'End of reaction' will cause the counting of the integrator to stop.

### Command Part

The command part is also modular. It consists of a program transmitter and a programmable controller. Management of the process control takes place through dialogue between these modules.

We use a programmable controller in which it is possible to write one to three sequential programs and one combination program. The programming of the combination part is a direct translation of a ladder diagram based on Boolean algebra. The program is constantly scrutinized and, following the logical state of the inputs, the outputs are activated or deactivated. The programming of the sequencer part is a translation of the functional diagram.

The program is an association of stage modules; its evolution is as follows: the stage modules are active one after the other; the active stage module issues the signal commanding the actions associated with the stage; a stage module becomes active when the preceding module is active and the condition of transition called receptivity is verified; and when the stage module becomes active, the previous module is deactivated.

The installation of the automated system is shown in figure 2 and the symbols used are explained in table 1.

### Automation of indalpine preparation

The above system was used to automate the catalytic hydrogenation of 3-[2-(4-pyridyl)ethyl]indole hydrochloride. The aim of this hydrogenation was to study the influence of temperature and pressure on the preparation of indalpine in order to optimize the reaction.

### *Reaction*

The hydrogenation reaction of 3-[2-(4-pyridyl)ethyl]indole hydrochloride takes place in hydrochloric acid medium in the presence of platinum on carbon as a catalyst:

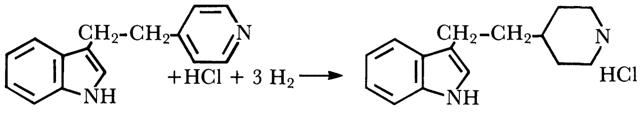

This is a slightly exothermic reaction and, in the case of important hydrogenation, leads to the formation of surhydrogenated products.

### *Operating mode*

The catalyst is loaded into the reactor and covered with distilled water. The manual operating mode is structured in GRAFCET form. From the first level GRAFCET (shown in figure 3) a so-called second-level GRAFCET is

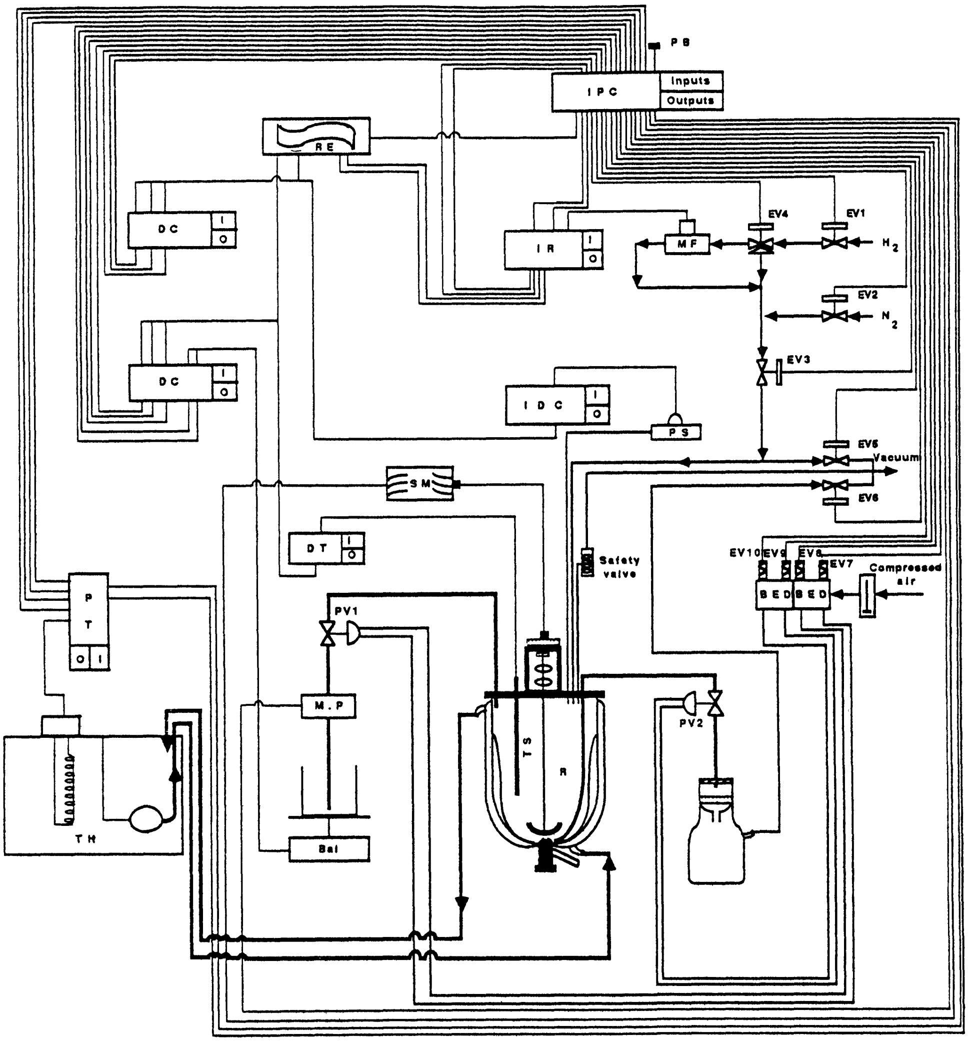

*Figure 2. Installation of the automated pressurized modular liquid–gas reactor.*

established [3]. This must take into account the technological means used to carry out the different operations. It will therefore be closely linked to the sensors and actuators used.

The programs from the various automated control appliances will therefore be a translation of this GRAFCET.

*Programming the programmable controller*

The programming of the programmable controller is carried out in two parts:

1. A sequencer part, in which programming comes directly from the GRAFCET. The sequencer takes over the longest branch of the GRAFCET. As in any computer program, it is only possible to return to a previous step if the order is explicitly given. The program consists of a series of stages, each stage consisting of its address, the operation to be carried out, 'Operand' data and outputs. The passage from one stage to the following stage is sanctioned by the validation of transition.

2. An automated part in which programming is a direct translation of the ladder diagram based on Boolean

*Table 1. Abbreviations used in figure 2.*

| Abbreviation | Designation |
|---|---|
| Bal | Balance with analogue output |
| BED | Bistable electropneumatic distributor |
| DC | Digital comparator |
| DT | Digital thermometer |
| MP | Electromagnetic metering pump |
| EV | Electrovalve |
| IDPC | Indicator and digital conditioner |
| IPC | Industrial programmable controller |
| IR | Integrator |
| MF | Mass flow meter |
| PV | Pneumatic valve |
| PB | Press button |
| PS | Pressure sensor |
| PT | Program transmitter |
| R | Reactor |
| RE | Recorder |
| SM | Stirring motor |
| TS | Temperature sensor |
| TH | Thermostatic circulation bath |

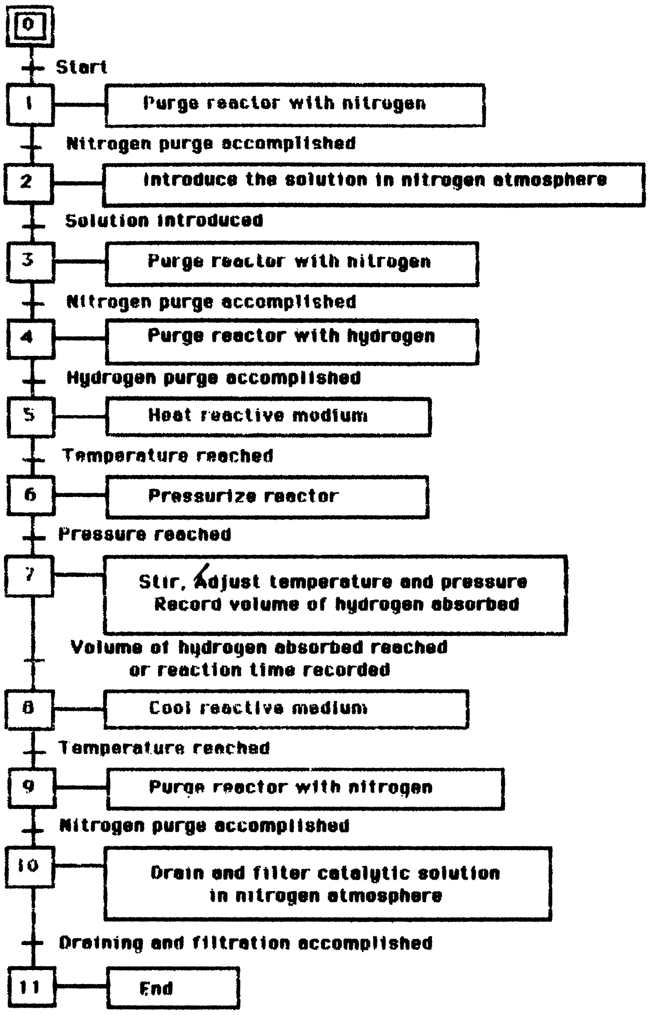

*Figure 3. First level GRAFCET.*

algebra. This programmable controller takes simultaneous changes into consideration. The program is continuously examined and, according to the logical state of the inputs, the outputs are activated or deactivated.

The synchronization between the two parts is carried out by calling on the number of the sequence step in which these operations must be carried out.

*Programming the program transmitter*

The program transmitter is programmed in segments. The evolution of the programmed variable is strictly linear. The temperature variation curve on a time scale is decomposed into low-order segments.

The operating mode takes into account numerous temperature variations according to the profile given in figure 4. It is therefore necessary to be able successively to heat or cool the reactive medium. The program transmitter supplies operating thresholds based on time. It also has logical inputs and outputs corresponding to those of the programmable controller. This is why, where the length of operations is concerned, we have left the calculations either to the programmable controller or to the program transmitter. This sends a logical signal, at the end of the segment, to the input of the programmable controller.

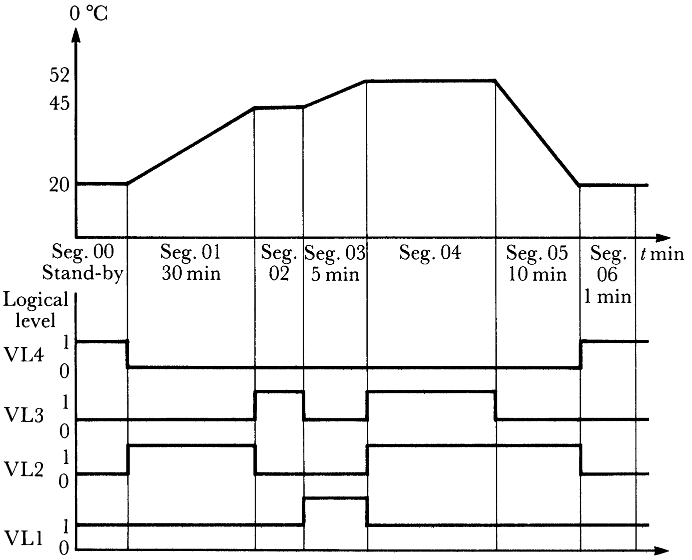

Segment 00: Stand-by position to be kept at 20 °C. No time to be programmed
Segment 01: Rise from 20 °C to 45 °C in 30 min.
Segment 02: Constant at 45 °C.
Segment 03: Rise from 45 °C to 52 °C in 5 min.
Segment 04: Constant at 52 °C
Segment 05: Fall from 52 °C to 20 °C in 10 min.
Segment 06: Constant at 20 °C for 1 min.

*Figure 4. Profile of temperature and logical level of outputs of the program transmitter.*

However, it is sometimes necessary to regulate the temperature for an undetermined time when the end of this regulation is indicated by an event other than time, amount of reagent introduced, temperature of the reactive medium, etc. In this case, the program transmitter is blocked at the desired temperature using the logical input of a program stop.

Control of the temperature of the thermostatically controlled bath is effected. In order to continue the remainder of the program, a check is made that the temperature of the reactive medium has reached the desired threshold. (Before this, we carried out trials by regulating the temperature of the reactive medium in relation to that of the thermostatically controlled bath.)

*Execution of the automated manipulation*

Before the programmable controller can take over the system, the following manual operations must be carried out:

(i) Load the catalyst into the reactor and make sure it is hermetically sealed.

(ii) Switch on the electrical power supply, the programmable controller and the thermostatically controlled bath.

(iii) Fill the reagent storage tank.

(iv) Prime the electromagnetic metering pump which introduces the reagents.

(v) Display the various thresholds for the comparators (pressures, temperatures and the volume of reagent to be introduced) and for the integrator (flow, reaction time and volume of hydrogen to be introduced).

(vi) Display the speed for unwinding the paper and, for each channel, the range of measurements of the recorder.

(vii) Open the water and compressed air taps in order to supply, in succession, the water pump to create a vacuum and the electropneumatic distributors in order to operate the pneumatic valve.

(viii) Open the nitrogen and hydrogen bottles and adjust the pressure to slightly higher than that in the experiment.

(xi) Set the program transmitter to 'AUTOMATIC' so that the program for each channel is in the '00' segment which corresponds to the stand-by position of the apparatus.

At the end of these operations, set the programmable controller to 'MONITOR.' From this moment, the control system takes over all the operations and the automated reaction is carried out according to the second-level GRAFCET.

By pressing the start-button, automatic manipulation is set in motion. The first operation is to make sure that the pneumatic valves are closed by activating electrovalves EV8 and EV10 of electropneumatic distributors 1 and 2 for 0.5 s.

Next, in order to introduce the reagents into a nitrogen atmosphere, the purge of the reactor with nitrogen is carried out by creating a vacuum and introducing nitrogen. The vacuum is obtained by activating electrovalve EV5. When the pressure $P_0$ = 80 mbar (corresponding to the vacuum) is reached; the nitrogen is introduced by opening electrovalve EV2 until the required $P_1$ = 1 bar of nitrogen pressure is reached.

Next, the reagent is introduced. For this pneumatic valve VP1 is opened by the pressure of compressed air sent through channel 1 of electropneumatic distributor '1' by activating electrovalve EV7 for 0.5 s and then by operating the electromagnetic metering pump until the required weight of reagent has been reached.

After introduction of the reagent, pneumatic valve VP1 is closed by operating electrovalve EV8 for 0.5 s. Once this time has elapsed, the system remains on stand-by until the temperature $\theta_1$ = 20 °C in the reaction medium is reached. This temperature corresponds to the '00' stand-by position of the program transmitter.

Next, the nitrogen purge is carried out twice as described above. After each purge with nitrogen, the counter $CNT_1$, is decremented by one unit.

The purge of the reactor with hydrogen is carried out after the lurge with nitrogen. In order to do this, a vacuum is created in the reactor and electrovalve EV5 is switched on until the point $P_0$ is reached, then hydrogen is introduced into the reactor and electrovalve EV1 is opened until the pressure $P_1$ is reached. The purge with hydrogen is repeated three times. At the end of each purge the counter $CNT_2$ is decremented by one unit.

Next, the program transmitter is operated in order to transmit the first segment of its program by activating input EP1 and simultaneously inputs EP1 and EP2 for 0.6 s each. These two inputs remain active until the logical output VL3 of the transmitter reaches the upper level. This logical output corresponds to the end of segment 01.

Then, the program transmitter is blocked on stand-by at the end of the first segment by activating input EP2 until a temperature of the reaction medium of $\theta_2$ = 45 °C has been reached. Once this temperature has been attained, hydrogen is introduced into the reactor and electrovalve EV1 is opened until the pressure $P_2$ = 2.7 bar has been reached. At the same time, the program transmitter is blocked on the segment.

When the recommended pressure $P_2$ has been reached, the program transmitter is again activated to set in motion the second and third segments of its program. The end of segment 03 corresponds to logical outputs VL2 and VL3.

Next, the program transmitter is blocked on stand-by until the recommended temperature $\theta_3$ = 52 °C has been reached. Once this temperature is obtained, the program transmitter is blocked while the pressure is being balanced in relation to the rise in temperature in order to maintain it at the experimental pressure. Thus, if the pressure is higher than the recommended $P_3$ = 3 bar, a vacuum is created in the reactor until the recommended $P_3$ is at the lower level. If the pressure is below this recommended level, hydrogen is introduced until the desired level $P_3$ reaches the higher level. After each of these operations a waiting time of 5 s enables the pressure read by the sensor to be stabilized. This cycle is repeated twice and at the end of each cycle counter $CNT_3$ is decremented by one unit.

When the pressure is balanced and equal to the experimental pressure, the programmable controller starts the integration process by activating the 'Integration' input IR2 of the digital integrator. The program transmitter is still blocked.

The 'macro-stage' following the start-up of integration corresponds to the reaction of hydrogenation itself. Thus, the mass flow meter is put in a measuring position by activating electrovalve EV3 to force the hydrogen through the flowmeter; the recorder and the stirring motor are set in motion. As the program transmitter is blocked, the temperature of the reaction medium is regulated.

The hydrogenation reaction at the same time as stirring begins. The reaction medium is maintained at a pressure $P_3$ by means of 'ON/OFF' regulation of hydrogen introduction by electrovalve EV1. If the flow-rate of

*Table 2. Operating conditions and responses.*

| Trial No. | Temperature (°C) | Time (min) | Pressure (bar) | Response | Standard deviation | Square of error |
|---|---|---|---|---|---|---|
| 1 | 52 | 105 | 3 | 57·4 | −1·04 | 1·08 |
| 2 | 52 | 105 | 3 | 58·8 | +0·36 | 0·13 |
| 3 | 52 | 105 | 3 | 58·3 | −0·14 | 0·02 |
| 4 | 52 | 105 | 3 | 59·7 | +1·26 | 1·59 |
| 5 | 52 | 105 | 3 | 58·0 | −0·44 | 0·19 |
| | | | | Av. 58·44 | | Σ3·01 |

Degrees of freedom (df) = 4. $\sigma^2 = \Sigma/\mathrm{df} = 3{\cdot}01/4 = 0{\cdot}75$.

hydrogen or the pressure exceed the recommended levels, the programmable controller activates the output connected to the logical input IR1 'Security' of the integrator, which stops integration during this period.

Once the amount of hydrogen needed for the reaction has been introduced or the time fixed for the reaction has expired (the end of the 'macro-stage'), the program transmitter is reactivated until the VL2 logical output is at the low level and the logical output VL4 is at the high level. The program transmitter is then blocked until the temperature $\theta_1 = 20\,°C$ of the reaction medium is reached.

Next, the whole system is purged with nitrogen in order to eliminate any trace of hydrogen, by creating a vacuum in the system through activation of electrovalve EV5 and then introducing nitrogen through electrovalve EV2. This nitrogen purge is repeated three times and at the end of each cycle the counter $CNT_4$ is decremented by one unit.

Once the nitrogen purge has been completed a vacuum is created in the filtering system by activating electrowave EV6 for 5 min. This electrovalve remains active during draining and filtering.

Finally, draining and filtering of the catalytic solution in a nitrogen atmosphere are carried out simultaneously. In order to drain off by aspiration, pneumatic valve VP2 is opened by activating electrovalve EV9 and then closed by operating electrovalve EV10. Each operation lasts 0.5 s. It takes 10 s to filter the amount drawn off. We repeated this cycle 30 times, because of the limited capacity of the plate funnel made of fritted glass, in order to empty the reactor completely.

Between two successive cycles, the pressure of nitrogen is re-established in the reactor by activating electrovalve EV2 until the desired pressure $P_1$ is reached. At the end of each cycle the counter $CNT_5$ is decremented by one unit.

### Results

The operating conditions, time and pressure and the responses obtained, the experimental efficiency, standard deviation and the square of the errors are given in table 2.

We carried out five automated experiments under identical operating conditions to be certain that the apparatus was functioning properly and that the results were reproducible.

### Conclusion

We have described the setting-up of an automated modular liquid–gas reactor under moderate pressure in order to carry out heterogeneous catalysis reactions involving at least one gaseous reagent and one liquid reagent. Pressure can be regulated by a sensor. The use of a special reactor makes work possible at an absolute pressure of 5 bar. Introduction of liquid reagent is automated by means of apparatus consisting of a pneumatic valve, an electropneumatic metering pump and a balance. Gaseous reagents may be introduced by using a set of electrovalves. This system enables a series of reactor purges to be carried out. Stirring is mechanical. The arm of the stirrer is driven by a magnetic coupling system designed to ensure that the reactor remains perfectly sealed under the maximum pressure used. The temperature of the reaction medium is regulated and controlled by a thermostatic circulating bath linked to a digital program transmitter. Gas flow is controlled and measured by a mass flow meter. The use of a digital integrator makes it possible to measure the volume of gas absorbed and to trace curves related to flow time and volume of gas. Draining of the catalytic solution is carried out by aspiration using a pneumatic valve and an electrovalve linked to a water pump. Finally, the whole set-up is controlled by a programmable controller.

This system provides the chemist with the possibility of studying gas absorption curves, i.e. studying the relationship between the speed of absorption of gas and the nature of solvent, catalytic reagents, temperature, etc., an improvement in the yield of products, stabilization of yields and quality, at the highest level, through a more accurate parametric procedure, better reproducibility of operations and reliability of trials, a means of reproducing industrial conditions in the laboratory, thus making transfer from laboratory to workshop easier, the possibility of putting experimental designs into operation and optimizing syntheses and a means of carrying out chemical manipulation in complete safety.

### References

1. Legrand, M. and Foucard, A., *Journal of Chemical Education*, **55** (1978), 767.
2. Porte, C., Roussin, D., Bondiou, J.-Cl. and Delacroix, A., *Bulletin de la Société Chimique de France*, **9** (1987), 166.
3. Blanchard, M., *Comprendre Maîtriser et Appliquer le GRAFCET (CEPADUES, Toulouse, 1979).*

